# The role of circulating miRNAs in the diagnosis of osteoporosis miRNAs in osteoporosis

**DOI:** 10.1590/1806-9282.20231724

**Published:** 2024-09-16

**Authors:** Senay Balci, Nurdan Orucoglu, Didem Derici Yildirim, Cagri Eroglan, Özlem Bolgen Cimen, Lulufer Tamer, Mehmet Burak Yavuz Cimen

**Affiliations:** 1 Mersin University, Medical Faculty, Department of Medical Biochemistry – Mersin, Turkey.; 2 Mersin University, Medical Faculty, Department of Rheumatology – Mersin, Turkey.; 3 Mersin University, Medical Faculty, Department of Biostatistics – Mersin, Turkey.; 4 Mersin University, Medical Faculty, Department of Physical Medicine and Rehabilitation – Mersin, Turkey.

**Keywords:** Osteoporosis, Osteopenia, miRNAs, miR-210, Bone density

## Abstract

**OBJECTIVE:**

Osteoporosis, defined as a systemic skeletal disease, is characterized by increased bone fragility and fracture risk. Studies have shown that dysregulation of the functions of miRNAs or the mechanisms they mediate may be an important pathological factor in bone degeneration. Therefore, the aim of the study was to determine the role of miRNAs, which are thought to play a role in bone metabolism, in osteoporosis.

**METHODS:**

The study included 48 patients who were diagnosed with osteoporosis according to the results of a bone mineral density assessment by quantitative computed tomography and 36 healthy individuals. MiRNAs from plasma samples obtained from blood samples taken into ethylenediaminetetraacetic acid (EDTA) tubes were isolated with the miRNA isolation kit and converted to cDNA. Expression analysis of miR-21-5p, miR-34a-5p, miR-210, miR-122-5p, miR-125b-5p, miR-133a, miR-143-3p, miR-146a, miR-155-5p, and miR-223 was performed on the real-time PCR (RT-PCR) device.

**RESULTS:**

When miRNA expression levels in the patient group were compared with the control group, all miRNAs were found to be downregulated in the patients. When fold changes in expression levels in the patient group were examined, significant differences were found in miR-21-5p, miR-133a, mir143-3p, miR-210, and miR-223. In the receiver operating curve analysis, area under the curve=0.882 for the combination of miR-34, miR-125, miR-133, and miR-210.

**CONCLUSION:**

In this study, it was determined that the combined effects of miRNAs, as well as their single effects, were effective in the development of osteoporosis. Therefore, a miRNA panel to be created can make a significant contribution to the development of novel diagnostic and treatment approaches for this disease.

## INTRODUCTION

Osteoporosis is a disease characterized by low bone mass and deterioration of bone architecture, resulting in decreased bone strength and increased fracture risk. The World Health Organization defines osteoporosis using bone mineral density (BMD) and the T score. The T score is expressed as the mean BMD of a healthy young adult, or as a standard deviation (SD) of how much a result differs from the mean. A T score of "0" indicates that BMD is equal to the norm for a healthy adult. The higher the SD values below 0, indicated as negative numbers, the lower the BMD and the higher the fracture risk. While osteoporosis is defined as a T score of <-2.5, osteopenia or low bone density is defined as a T score between -1.0 and -2.5^1^.

Osteoporosis, defined as a skeletal disorder characterized by weakened bone strength, is considered a silent disease. For this reason, it is recommended to screen, especially in individuals over the age of 65 years, taking into account the risk factors^2^. Studies have shown that miRNAs, which are involved in many biological processes, regulate gene expression that controls osteoblast-related bone formation and osteoclast-related bone remodeling and also play a role in mechanisms such as osteoclast differentiation and osteoblast–osteoclast communication^3-8^.

MiRNAs are a class of non-coding RNAs about 18–25 nucleotides in length, and they control gene expression at the post-transcriptional level by providing epigenetic modification. It is thought that up to 60% of human protein-coding genes can be regulated by miRNAs. They bind to the 3-untranslated regions (3-UTR) of target genes, causing mRNA degradation and inhibition of transcription. MiRNA regulation processes are complex because each miRNA binds to multiple targets, and several miRNAs target the same mRNA^3-7,9^.

Studies demonstrate that epigenetic modifications are effective in the development of osteoporosis. However, these mechanisms that play a role in osteoporosis have not been fully determined. Therefore, this study aimed to determine the role of miRNAs, which are important epigenetic regulators that affect many biological processes, including bone metabolism, and play a role in the control of gene expression, in osteoporosis.

## PATIENTS AND METHODS

The study consisted of a patient group (n=48) and a healthy control group (n=36). The patients were divided into two subgroups based on BMD evaluation results by quantitative computed tomography (qCT): 17 patients diagnosed with osteopenia and 31 patients diagnosed with osteoporosis. The mean age of the participants included in the study was 40 years in the control group and 60 years in the patient group, and their examination and diagnosis were carried out by the Department of Physical Therapy and Rehabilitation. Participants with a diagnosis of cancer and any systemic disease were excluded from the study.

This study was approved by the University Clinical Research Ethics Committee (Approval No:2019/276). Written consent was obtained from all participants.

In this study, 10 miRNAs (miR-21-5p, miR-34a-5p, miR-210, miR-122-5p, miR-125b-5p, miR-133a, miR-143-3p, miR-146a, miR-155-5p, and miR-223) that play a role in bone metabolism were analyzed. miRNAs were identified using the "miR2Disease," "mirbase," and PubMed (MEDLINE) databases. For miRNA analysis, venous blood samples were taken from 84 participants in the study in an ethylenediaminetetraacetic acid (EDTA) tube and then centrifuged at 2,000 *g* for 10 min. The plasma obtained after centrifugation was taken into a sterile microcentrifuge tube and centrifuged again. Plasma samples obtained after repeated centrifugation were stored in a deep freezer at -80°C.

MiRNAs from plasma samples were isolated with a miRNA isolation kit (Roche Diagnostics, GmbH, Mannheim, Germany) and converted to cDNA. The obtained cDNAs were measured with nanodropper before the PCR step.

Expression analysis of 10 target miRNAs was performed on the real-time PCR (RT-PCR) device (Roche LightCycler 480). To amplify cDNAs in terms of the reference gene (snord) and to mark the relevant regions, BrightGreen Master Mix, and miRNA PCR primer mixes were prepared according to the specified volumes, following the manufacturer's recommendations, and real-time PCR was processed.

Relative expression analyses of miRNAs were calculated by the comparative ΔCT (ΔΔCT) method. Fold change (FC) was calculated with the equation 2-^ΔΔCt10^.

## STATISTICAL METHODS

The p-values are calculated based on a Student's t-test of the replicate 2-^ΔCt^ values for each gene in the groups. The diagnostic power of the miRNAs (FCs) was analyzed with the receiver operating curve (ROC). Multiple logistic regression analysis was used to calculate area under the curves (AUCs) for gene combinations. For the combinations, the AUC was higher than 0.80, and the diagnostic power was "good." Statistica v.13.3 package program and MedCalc v.10.3 were used to perform statistical analysis. The level of significance was set at p<0.05. The covariance effect of age in group comparisons was tested with covariance analysis.

## RESULTS

In terms of the BMD measurement regions of 48 patients (age: 60.46±8.951 years) included in this study, 37 patients were found to be from the lumbar region and 11 patients from the femur region. While the mean BMD value was 46.11±28.33 and the T score was -4.00±0.773 in osteoporosis patients, it was determined that the BMD value was 78.86±35.73 and the T score was -2.62±0.574 in osteopenia patients.

The mean age of healthy individuals in the control group was determined to be 39.55±11.416 years. It was tested by covariance analysis that the age difference between the two groups did not have a statistical effect on the miRNA expression levels between the groups (p>0.05).

### Data on miRNA expression levels

When miRNA expression levels in the patients (osteoporosis+osteopenia) were compared with the control, all miRNAs were found to be downregulated in the patient group. When fold changes in expression levels in the patient group were examined, significant differences were found in miR-21-5p (FC=0.339; p=0.016), miR-133a (FC=0.085; p=0.017), mir143-3p (FC=0.095; p=0.025), miR-210 (FC=0.067; p=0.001), and miR-223 (FC=0.155; p=0.001) (Table 1).

**Table 1:** Fold changes of miRNAs in the patient group compared to the control group.

miRNA	2^-ΔCt^		Fold change	p
	Control	Patient		
mir21-5p	0.090	0.030	0.339	0.016
mir 34a-5p	0.128	0.014	0.108	0.119
mir122-5p	0.051	0.014	0.275	0.075
mir125-5p	0.943	0.599	0.636	0.103
mir133a	0.408	0.035	0.085	0.017
mir143-3p	0.102	0.010	0.095	0.025
mir146	0.479	0.025	0.053	0.091
mir155-5p	0.056	0.015	0.261	0.111
mir210	8.201	0.549	0.067	0.001
mir223	0.261	0.041	0.155	0.001

The patients were divided into two subgroups, consisting of osteoporosis and osteopenia patients. When miRNA expression levels of the two groups were compared with the control group, it was found that miR-21-5p (FC=0.334; p=0.036), miR-34a-5p (FC=0.113; p=0.12), miR-122-5p (FC=0.302; p=0.02), miR-133a (FC=0.094; p=0.022), miR-210 (FC=0.061; p=0.005), and miR-223 (FC=0.172; p=0.001) were significantly downregulated in osteoporosis. In osteopenia, miR-155-5p (FC=0.224; p=0.045) and miR-223 (FC=0.131; p=0.002) were found to be significantly downregulated (Table 2).

**Table 2:** miRNA expression levels in osteoporosis and osteopenia patients compared to the control group.

miRNA	Osteoporosis			Osteopenia		
	2^-ΔCt^	Fold change	p	2^-ΔCt^	Fold change	p
mir21-5p	0.030	0.334	0.036	0.031	0.348	0.135
mir 34a-5p	0.014	0.113	0.012	0.013	0.099	0.760
mir122-5p	0.015	0.302	0.020	0.012	0.231	0.610
mir125-5p	0.602	0.639	0.079	0.594	0.630	0.358
mir133a	0.038	0.094	0.022	0.029	0.071	0.144
mir143-3p	0.011	0.104	0.071	0.008	0.080	0.188
mir146	0.021	0.043	0.128	0.036	0.075	0.411
mir155-5p	0.016	0.283	0.331	0.013	0.224	0.045
mir210	0.500	0.061	0.005	0.653	0.080	0.058
mir223	0.045	0.172	0.001	0.034	0.131	0.002

### ROC analysis data

ROC analysis was performed using the miRNA expression levels of the patient and control groups. When the AUC of the combinations of different miRNAs was evaluated, it was determined that especially miR-210 increased the diagnostic power among those with an AUC >0.80. The AUC of the combination of miR-34a-5p, miR-125, miR-133a, and miR-210 was found to be 0.882 (sensitivity: 93.7; specificity: 71.4), and it was evaluated as the combination with the highest diagnostic power (Table 3).

**Table 3:** ROC analysis results.

miRNA	AUC	SE	p
34+125+133+210*	0.882	0.036	<0.001
mir_34+mir_210	0.807	0.048	<0.001
mir_125+mir_210	0.837	0.044	<0.001
mir21+mir34+mir210	0.814	0.047	<0.001
mir34+ mir122+mir210	0.826	0.045	<0.001
mir122+mir125+mir210	0.848	0.042	<0.001
mir125+mir133+mir210	0.856	0.041	<0.001
mir146+mir210+mir223	0.799	0.047	<0.001
mir21+mir34+mir122+mir210	0.812	0.047	<0.001
mir34+ mir122+mir125+ mir210	0.873	0.037	<0.001
mir122+mir125+mir133+mir210	0.852	0.042	<0.001
mir125+mir133+mir143+mir210	0.856	0.041	<0.001
mir146+mir155+mir210+mir223	0.824	0.044	<0.001
mir21+mir34+mir122+mir125+mir210	0.863	0.039	<0.001
mir34+mir122+mir125+mir133+mir210	0.877	0.037	<0.001
mir122+mir125+mir133+mir143+mir210	0.854	0.041	<0.001
mir125+mir133+mir143+mir146+ mir210	0.856	0.041	<0.001
mir133+mir143+mir146+ mir155+mir210	0.803	0.047	<0.001
mir143+mir146+ mir155+mir210+mir223	0.827	0.044	<0.001
mir21+mir34+mir122+mir125+mir133+mir210	0.863	0.040	<0.001
mir34+mir122+mir125+mir133+mir143+mir210	0.876	0.037	<0.001
mir122+mir125+mir133+mir143+mir146+mir210	0.852	0.042	<0.001
mir125+mir133+mir143+mir146+mir155+mir210	0.861	0.040	<0.001
mir133+mir143+mir146+mir155+mir210+mir223	0.845	0.041	<0.001
mir21+mir34+mir122+mir125+mir133+mir143+mir210	0.863	0.040	<0.001
mir34+mir122+mir125+mir133+mir143+mir146+ mir210	0.876	0.037	<0.001
mir21+mir34+mir122+mir125+mir133+mir143+ mir146+mir210	0.862	0.040	<0.001
mir34+mir122+mir125+mir133+mir143+mir146+mir155+mir210	0.879	0.036	<0.001
mir122+mir125+mir133+mir143+mir146+mir155+mir210+mir223	0.854	0.040	<0.001
mir21+mir34+mir122+mir125+mir133+mir143+ mir146+mir155+mir210	0.858	0.040	<0.001
mir34+mir122+mir125+mir133+mir143+ mir146+mir155+mir210+mir223	0.867	0.038	<0.001
mir21+mir34+mir122+mir125+mir133+mir143+ mir146+mir155+mir210+mir223	0.863	0.040	<0.001

* The combination of miR-34a-5p, miR-125, miR-133a, and miR-210 was evaluated as the combination with the highest diagnostic power.

## DISCUSSION

Bone metabolism is a delicately balanced process involving bone formation and bone resorption, mediated by osteoblasts and osteoclasts. Osteoporosis, which occurs as a result of increased bone destruction and decreased formation, is characterized by a decrease in bone mass and deterioration in the microstructure of bone tissue. The disease is associated with an increased risk of fracture, and this also affects the quality of life of patients^11^. In osteoporosis, many factors, including genetic and environmental factors, affect osteoblast and osteoclast differentiation and activity. The incidence of osteoporosis increases markedly with the aging of the population^12^. It is stated that epigenetic regulators such as miRNAs, which play a role in many cellular processes and in the control of gene expression, may be effective in the epigenetic mechanism of osteoporosis, which is a multifactorial disease that is still not fully determined^13,14^. In this study, it was aimed at determining the role of miRNAs in osteoporosis and creating a miRNA panel that can be used as a diagnostic and/or screening test in line with the data obtained. For this purpose, the expression levels of 10 determined miRNAs were examined, as was the downregulation of mir21-5p, miR-34a-5p, miR-122-5p, miR-133a, miR-210, and miR-223 in osteoporosis patients. It was found that miR-155-5p and miR-223 were downregulated in osteopenia.

While miR-21 was found to be increased in the serum and bone tissue of osteoporotic patients, its expression level was found to be significantly decreased in osteoporotic and osteopenic women with vertebral fractures^15,16^. Through a positive feedback loop involving programmed cell death, miR-21 is regulated by the osteoclastogenesis factor c-Fos, and subsequently, it has been reported to promote RANKL (receptor activator of NF-κB ligand)-mediated osteoclastogenesis^17^. In a study by Huang et al., it was shown that miR-21-5p is downregulated in the process of osteoclast differentiation, and miR-21-5p may have an effect on osteoclast differentiation through S-Phase Kinase-Associated Protein 2^18^.

It is stated that miR-34a-5p, one of the microRNAs involved in osteogenic differentiation, has RUNT-associated transcription factor 2 (RUNX2) as its target gene. It has been reported that miR-34a-5p induces osteogenic differentiation of BMSCs and increases bone metabolism by targeting HDAC1 to activate ER-a^19^.

In a study conducted on patients with low BMD, it was reported that miR-122-5p was downregulated. It has been shown that miR-122-5p is associated with mRNAs expressed in osteoblast or osteoclast cells, and these mRNAs target complementary sequences encoding proteins that have been associated with osteoporosis. The target genes—bone morphogenetic protein inducible kinase, follicle-stimulating hormone beta subunit, RUNX233, and vitamin D receptor—have been specifically associated with human osteoblasts and osteoclasts^20^.

In early osteogenesis, it has been reported that BMP-2 signals downregulate miR-133 and miR-135, which suppress two transcription factors involved in osteogenesis, RUNX2 and SMAD5, by forming a transcriptional complex^21^. When miR-133a is overexpressed, it targets the RUNX2 gene 3-UTR and suppresses alkaline phosphatase production and thus osteoblast differentiation. Cheng et al. also noted that miR-133a promotes bone resorption and could potentially inhibit bone formation^22^.

MiR-155 and miR-223 are associated with both vascular calcification and osteoporosis^23^. TGFβ1/Smad4 signaling has been shown to affect osteoclast differentiation through the regulation of miR-155 expression. miR-223 has multiple roles in regulating bone metabolism. It exhibits antagonistic or synergistic functions at different expression levels in osteoclast differentiation. When miR-223 is upregulated during abnormal bone metabolism, the expression of IKKa and NFIA is downregulated, resulting in decreased osteoclast differentiation or enhanced osteoclast differentiation^24^.

In a study using an ovariectomized rat model, it was reported that miR-210 expression was significantly reduced in femoral tissue. High expression of miR-210 has been reported to improve the microstructure of bone tissue, regulate bone formation and resorption, and alleviate osteoporosis. Studies suggest that it may play these roles by activating the VEGF/Notch1 signaling pathway^25^.

Osteoporosis and osteoporosis-related fractures are common causes of morbidity and mortality in older adults. High BMI and increased risk of fragility fractures lead to deterioration in quality of life. In healthy adults, especially in women over the age of 65 years, screening is very important in terms of determining and applicability of the measures to be taken to prevent osteoporosis. Pharmacological treatments such as bisphosphonates are applied to diagnosed individuals. The choice of treatment is based on safety, cost, convenience, and other patient-related factors^8^.

Various RNAs associated with osteoporosis, such as miRNAs, target key genes and signaling pathways that affect the functions of osteoblasts and osteoclasts and play important roles in their development. Investigating these RNAs and understanding their interactions will contribute to a more comprehensive understanding of the pathogenesis of osteoporosis. As a result, it is anticipated that it will help develop more effective drugs and treatment strategies and ultimately provide a social benefit^26^.

In conclusion, in this study, it was determined that the combined effects of miRNAs, as well as their single effects, were effective in distinguishing between osteoporosis and osteopenia. A miRNA panel can be a screening test for the disease. It is predicted that, when supported by further studies, it may be an important biomarker in diagnosis and therefore in supporting treatment. Further studies can be conducted that include larger populations and identify different risk factors in different age groups.

## ETHICS COMMITTEE APPROVAL

This study was approved by the Mersin University Clinical Research Ethics Committee (Approval No:2019/276). Written consent was obtained from all participants.
